# The hypothalamic transcriptome reveals the importance of visual perception on the egg production of Wanxi white geese

**DOI:** 10.3389/fvets.2024.1449032

**Published:** 2024-09-20

**Authors:** Lei Yang, Changze Jia, Yanzhong Li, Yafei Zhang, Kai Ge, Deyong She

**Affiliations:** ^1^College of Biological and Pharmaceutical Engineering, West Anhui University, Lu’an, China; ^2^Animal Husbandry Development Center of Lu’ an City, Lu’an, China; ^3^Anhui Wanxi White Goose Seed Farm Co., LTD., Lu’an, China; ^4^Lu’ an Academy of Agricultural Sciences, Lu’an, China

**Keywords:** egg production, geese, hypothalamus, visual perception, transcriptome

## Abstract

Egg performance significantly impacts the development of the local goose industry. The hypothalamus plays an essential role in the egg production of birds. However, few potential candidate genes and biological functions related to egg production in geese have been identified in hypothalamus tissue. In this study, 115 geese were raised and observed for 5 months during the laying period. To understand the regulation mechanism of egg production, the hypothalamus transcriptome profiles of these geese were sequenced using RNA-seq. The hypothalamus samples of four high egg production (HEP) and four low egg production (LEP) geese were selected and collected, respectively. A total of 14,679 genes were identified in the samples. After multiple bioinformatics analyses, Gene Ontology (GO) annotations indicated that genes related to egg production were mainly enriched in biological processes of “response to light stimulus,” “sensory system development,” and “visual perception.” Six potential candidate genes (*PDE6C*, *RHO*, *MFRP*, *F2*, *APOB*, and *IL6*) based on their corresponding GO terms and interaction networks were identified. These identified candidate genes can be used as selection markers to improve the egg production of Wanxi white geese. Our study highlights how visual perception may affect the regulation of geese egg production.

## 1 Introduction

Goose is a multi-purpose winged animal. Goosekeeping can expand the choice of basic food raw materials, including abundant protein and fat, as well as highly nutritious goose eggs. Meanwhile, it also provides raw materials for the light industry, such as feathers and down, which are used in the manufacture of high-quality bedding, clothing insulation, and various textile products (1). However, compared with the chicken and duck industries, the goose industry developed slowly due to the poor egg production performance of most goose breeds (2). In particular, geese, as seasonally reproducing waterfowl, had the strongest tendency toward broodiness among all poultry, which further led to a decrease in egg production and reproductive efficiency (3). Therefore, the question of how to improve goose egg production has become a crucial problem for the development of the goose industry.

Improving the genetic potential of goose egg production is an important strategy for the development of the goose industry. The traditional breeding techniques are based on the long-term selection of egg production and egg rate (4). However, the egg production performance of geese is controlled by multiple genes and is greatly affected by the environment, which directly leads to the time-consuming, laborious, and poor effect of traditional breeding (5). For the last three decades, based on the characteristics of egg production performance, molecular-assisted breeding technology has been widely used in poultry reproduction breeding to save breeding time and effort (6). Currently, the identification of candidate genes at genome and transcriptome levels by a variety of high-throughput techniques has become the mainstream of poultry reproduction breeding (7–9). Nevertheless, there are still few relevant studies on geese. A number of transcriptomic analyses have been conducted on the hypothalamus of poultry with different laying performances (5, 9, 10). In geese, a previous study on Xinjiang Yili geese suggested that the HPG axis may play a key role in regulating egg production (11). Moreover, another study on Zi-geese found that the hypothalamus plays an important role in light regulation of egg production (12). Visual input in the hypothalamus affects the synthesis and secretion of melatonin, which is a key hormone regulating the reproductive activities of seasonal breeders. In seasonal breeding birds such as geese, appropriate light exposure can stimulate the hypothalamus to release gonadotropin-releasing hormone (GnRH), which, in turn, promotes the release of luteinizing hormone (LH) and follicle-stimulating hormone (FSH). These hormones act directly on the ovaries, promoting follicle maturation and egg production (13).

The reproductive activities of birds are mainly regulated by the hypothalamic–pituitary–gonadal (HPG) axis (14). Even insignificant differences in hypothalamic function may affect reproductive performance in poultry, including follicular formation, ovulation, oviposition, and brooding behavior (15). Egg production in birds is a complex process regulated by multiple endocrine and environmental factors. The hypothalamus plays a critical role in this regulatory network, acting as the primary integrative center for reproductive hormone signaling (16). In addition to GnRH, the hypothalamus also produces other neuropeptides and neurotransmitters, such as kisspeptin, neuropeptide Y (NPY), and dopamine, which modulate the release of GnRH and subsequently influence reproductive processes. For instance, kisspeptin has been shown to directly stimulate GnRH neurons, playing a crucial role in the onset of puberty and the regulation of fertility in birds (17).

Wanxi white goose is a unique and popular breed of poultry in Anhui province, China, which is characterized by LEP and high economic value of down and meat production. Wanxi white geese lay 20–25 eggs per year. The peak egg-laying period occurs during the winter season, from January to March (18). In the current study, a high-throughput transcriptome was used to compare the hypothalamic transcriptional profiles of Wanxi white geese with different egg production performances. Through PPI network analysis and GSEA analysis, the biological functional differences between Wanxi white geese with HEP and LEP were deeply analyzed, and the key genes affecting goose egg production performance were excavated. These data will contribute to further clarifying the molecular regulation mechanism of goose reproduction.

## 2 Materials and methods

### 2.1 Animal management and tissue collection

A total of 115 Wanxi white geese, including 92 female geese (300 days old) and 23 male geese (600 days old), were bred at Anhui Wanxi white goose conservation farm, in Lu’an, China. All geese were housed in individual pens (1♂ and 4♀ per pen) with playground fields. All geese were provided the same diet (Table 1) and free access to feed and water. Eggs were collected and recorded at 5:00 pm every day. According to the continuous and complete egg production records (the laying period is from December 2020 to May 2021), we selected four HEP and four LEP geese. The HEP Wanxi white geese laid 31, 33, 32, and 32 eggs, respectively. The LEP Wanxi white geese laid 12, 11, 11, and 10 eggs, respectively. The body weight of each goose showed no significant difference, and all geese were vaccinated simultaneously and in equal amounts. They exhibited good mental condition and were free from other confounding factors. Through careful observation, no bullying was found among the groups. The ganders had similar body weights, and no significant differences were found in sperm motility and quantity. Additionally, these geese had two previous egg-laying experiences. Only differences in egg production were observed. The F0 geese (parents of the geese in the study) were bred from a preexisting goose population within our farm. These geese were raised using a high-level flat-net rearing system, with a layer height of 3 meters. The enclosures were made entirely of wood, with plastic net mats laid at the bottom. Natural lighting and ventilation were provided. The geese in the study had free access to food and water. They were fed a corn–soybean meal diet, with a metabolizable energy level of 12.25 MJ/kg and a crude protein content of 16.30%. The selected geese were slaughtered, and the hypothalamus were collected. All tissues were washed with RNA-free water and then frozen in liquid nitrogen. All samples were labeled and stored at −80°C until they were used for the extraction of total RNA.

**Table 1:** Primer pairs designed for RT-qPCR analysis.

Primer name	Amplicon size (bp)	Forward primer (5′ → 3′)	Reverse primer (5′ → 3′)
*β-actin*	159	ACACTGTGCCCATCTACGAA	CCTTGATGTCACGCACGATT
*WNT2B*	109	GTGTGCAACAAGATGTCCCG	GGAACTTGCACTCGCACTTG
*FSD2*	170	AAAAGTCTGCCAGCAAGGAC	TCAAGCCTTCAGCCTCAGTC
*PDE6C*	94	TCAAAGTCCCAGCAGAGGTC	TTGAATCCATGCCGCCAGTT
*HRG*	122	CAGACGCCCACCACATTTTC	GGCGGTTATGTCGTCTACCA
*TBX15*	136	CAATGCCTGCGACAACAGAC	GGGTATGCCCATGGAAGCAA
*COL6A2*	144	TCCCCAGCACCATACATTCG	ACTCTTGCCGCTCATTTCCA
*DBH*	162	CGGGCACAGCTCGTATTTTG	AGGTAGTCCTTGGGGTCACA
*KCNJ13*	101	TCGCTGGAGACACAACTCAC	AGCCCCAGGACCATCTGTAT

### 2.2 Total RNA extraction

The total RNA was isolated from the hypothalamus of each sample using TRIzol Reagent (Invitrogen Life Technologies, Carlsbad, CA, United States) according to the instructions. The concentration and quality of RNA were detected by the Nanodrop ND-1000 spectrophotometer (Implen, Westlake Village, United States) and Agilent 2,100 Bioanalyzer (Agilent Technologies, Santa Clara, CA, United States) according to the manufacturer’s protocol. Eight RNA samples (4 μg) with high quality and concentration were used to construct the transcriptome libraries. The remaining RNA samples were stored at −80°C for subsequent validation experiments.

### 2.3 cDNA library construction and Illumina sequencing

After quality control of eight RNA samples, all mRNA samples were purified and cleaved into fragments, which were reverse-transcribed and synthesized cDNA. AMPure XP system (Beckman Coulter, Beverly, United States) was used to determine the cDNA fragment size, and approximately 250–300 bp was selected for library construction. The constructed library was qualified and evaluated by Agilent. Following cluster generation, cDNA libraries were constructed and sequenced on Illumina HiSeqTM 2,500 (Illumina, San Diego, CA, United States) in Oebiotech Bioinformatics Technology Co., Ltd., Shanghai, China. High-throughput sequencing was conducted according to the manufacturer’s instruction, and 125 bp/150 bp paired-end reads were generated. The average read depth for RNA sequencing was approximately 20 million reads per sample. The transcriptome sequencing results and location information are shown in Table 1.

### 2.4 RNA-seq analysis and differentially expressed genes (DEGs)

Raw reads were first handled by the internal R script. Then, clean reads were obtained by removing reads containing adapters or poly-N and reads of low quality from the raw reads. Meanwhile, Q20, Q30, and GC contents of clean reads were calculated. Clean reads were mapped to the goose reference genome GCF_000971095.1^1^ using the TopHat software package. TopHat was used to delete portions of each read based on the accompanying quality information and then these reads were mapped to the reference genome of the goose.

Gene expression levels were calculated based on its Fragments per Kilobase of transcript per Million mapped reads (FPKM) values using Cufflinks v1.2.1. DEGs between the HEP and LEP geese were analyzed using the DEseq2 package V3.11, and gene counts from each sample were normalized using the DESeq2, utilizing the BaseMean value to estimate expression levels. Differential expression fold changes were calculated, and the significance was tested using a negative binomial distribution test (*p* < 0.05). Differential genes were ultimately selected based on both the fold changes and the results of the significance tests. The quality of the data was evaluated using log-box graphs and PCA analysis.

### 2.5 Functional analysis and hierarchical clustering of GO and KEGG pathways

Functional analysis of GO terms and the KEEG pathways annotation were performed in Metascape^2^ online server for all DEGs (19). In all tests, the *p*-value is calculated using Benjamin-corrected modified Fisher’s exact test, and a *p-*value of <0.05 is considered to be statistically significant.

To further analyze the DEG function, all enriched GO terms were performed hierarchical clustering. GO terms were collected and grouped into clusters based on the similarity of their members, and sub-trees with a similarity of >0.3 were considered clusters. The most significant term in a cluster is selected to represent the cluster.

### 2.6 Protein interaction network analysis and module selection

The DEGs related to reproductive regulation were predicted by functional enrichment analysis. These selected DEGs were then constructed into protein–protein interaction (PPI) networks using the STRING protein interaction database.^3^ In the PPI network, each node represents a protein, and each edge represents the interaction between the two proteins. Differential PPI network data files were visually edited using Cytoscape software v3.4.1.^4^

The CytoHubba application in Cytoscape was used to analyze the key genes through four centrality methods, including EPC, closeness, betweenness, radiality, and MCC. The hub gene was chosen based on the intersection of the five algorithms, and a Venn diagram was generated using an online website.^5^ The molecular complex detection (MCODE) application in Cytoscape was performed to screen the hub module of the PPI network. The criteria setting of MCODE is degree cutoff = 2, node score cutoff = 2, k-core = 0.5, and maximum depth = 100. GO and KEGG functional enrichment analyses were performed for genes in the module.

### 2.7 Gene set enrichment analysis

All expressed genes were used for GSEA analysis.^6^ Gene sets are available from the Molecular Signatures Database (MSigDB, http://www.broad.mit.edu/gsea/msigdb/). The GSEA program was run according to the default parameters. GSEA first sequenced all expressed genes according to the significance of gene expression differences between the HEP and LEP groups. Then the enrichment score of each gene set was calculated using the whole sequence list, which reflected the distribution of each gene set in the sequence list. The significant enrichment of the gene set was screened with a nominal *p*-value of <0.05 and a normalized enrichment score of (NES) ≥ 1.

### 2.8 qRT-PCR verification

In order to verify the repeatability and accuracy of RNA-seq data, four upregulated DEGs and four downregulated DEGs were randomly selected for real-time fluorescence quantitative PCR (RT-qPCR) validation, respectively. *β*-actin was used as a housekeeping gene. The reaction conditions of RT-qPCR were as follows: 1 μL of first strand cDNA (400 ng/μl), 5 μL of two SYBR Premix Ex TaqTM II (TaKaRa), 0.5 μL (10 μM) of forward primer and reverse primer, and 3 μL of deionized water.

qRT-PCR was performed on the ABI 7500 Real-Time PCR System (Applied Biosystems, Cafeteria, United States). Thermocycling parameters used for qRT-PCR were as follows: 95°C for 10 min, 40 cycles at 95°C for 10 s, 60°C for 40 s, and 95°C for 15 s, followed by a melting curve from 60°C for 60 s, 95°C for 30 s, and 60°C for 15 s. Three repeated tests were performed for each sample. The specificity of RT-qPCR products was verified by agarose gel electrophoresis and melting curve analysis. Gene expression values were estimated using the 2 ^-ΔΔCt^ method and normalized using GADPH. All primers were designed using the Primer-BLAST tools.^7^ Primer sequences are shown in Table 1.

### 2.9 Statistical analysis

The SPSS v20.0 software package was used for statistical analysis. RNA sequencing and RT-qPCR were compared by performing the Student’s *T*-test after the confirmation of normal distributions for non-parametric analysis. The resulting values were represented by means ± SEM, and a *p*-value of <0.05 was considered statistically significant.

## 3 Results

### 3.1 An overview of RNA sequencing and transcriptome alignment

An average of 48.97 and 49.94 million raw reads were obtained from HEP and LEP geese, respectively. After filtering the low-quality sequences, a total of 47.84 (clean ratio: 97.68%) and 48.72 (clean ratio: 97.56%) million clean reads were obtained from HEP and LEP geese, respectively, and were used for further analysis. The GC content of all samples ranged from 48.21 to 48.67%. The percentage of the Q30 base was above 90.00% (Supplementary Table S1). In summary, the sequencing data were suitable for subsequent data analysis.

The total mapped ratio between the reads and the reference genome of all the samples ranged from 87.47 to 87.88%. The uniquely mapped ratio between the reads and the reference genome of all the samples ranged from 86.42 to 86.88% (Supplementary Table S2). The results showed that the transcriptome data were reliable and suitable for subsequent analysis.

### 3.2 Identification and analysis of DEGs

**Figure 1:**
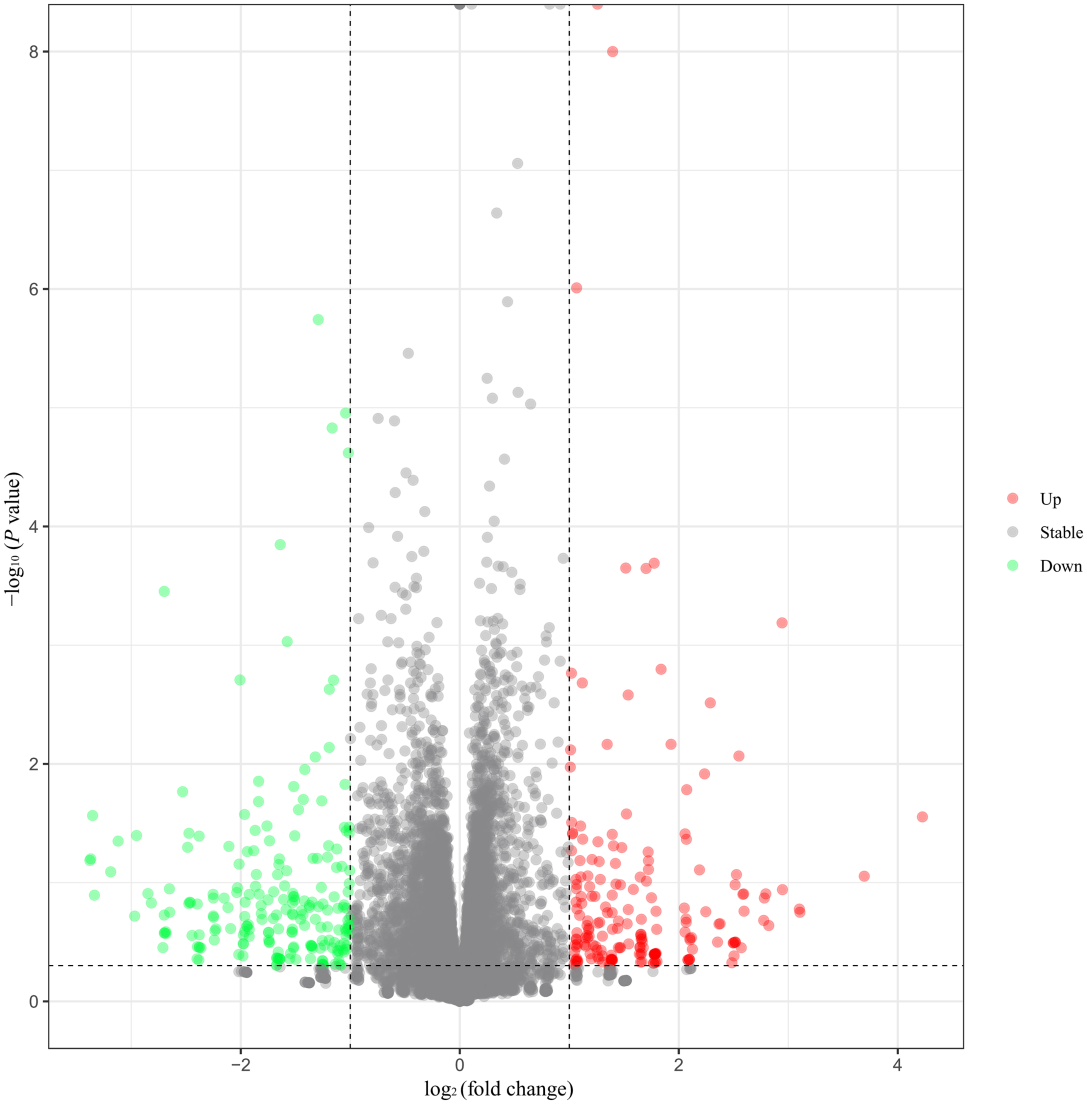
Volcano plot of differentially expressed genes (DEGs) in the hypothalamus. Note: Green spots indicate downregulated genes, and red spots represent upregulated genes. Gray spots represent genes that did not show obvious changes between the HEP and LEP samples.

A total of 14,679 genes were identified in the eight hypothalamus cDNA libraries. Among all the genes, 404 DEGs were identified, including 191 upregulated and 213 downregulated genes in the hypothalamus tissues of HEP geese compared to those of LEP geese (Figure 1). The top 10 upregulated and downregulated DEGs in the hypothalamus of HEP geese, ranked by log2 (fold change), are listed in Table 2. The most altered genes in HEP geese were *PDE6C* (upregulated, log_2_ (fold change) = 4.23, *p*-value = 2.79E-02) and *LOC106046287* (downregulated, log_2_ (fold change) = −3.38, *p*-value = 6.50E-02).

**Table 2:** The top 10 upregulated and downregulated DEGs in the hypothalamus of HEP geese.

Gene symbol	Log2FC^1^	*P*-value	Description	Regulation
*PDE6C*	4.23	2.79E−02	Phosphodiesterase 6C	Up
*LOC106049719*	3.69	8.82E−02	Coiled-coil domain-containing protein 81-like	Up
*CHRNA10*	3.11	1.78E−01	Cholinergic receptor nicotinic alpha 10 subunit	Up
*ODF1*	3.10	1.67E−01	Outer dense fiber of sperm tails 1	Up
*LOC106046796*	2.95	1.15E−01	SCO-spondin-like	Up
*LOC106049005*	2.94	6.48E−04	Retinol dehydrogenase 3-like	Up
*LOC106042972*	2.82	2.30E−01	Cytosolic phospholipase A2 epsilon-like	Up
*LOC106036800*	2.79	1.24E−01	Solute carrier family 22 member 13-like	Up
*TMEM238*	2.78	1.35E−01	Transmembrane protein 238	Up
*LOC106046797*	2.78	2.08E−01	SCO-spondin-like	Up
*LOC106046287*	−3.38	6.50E−02	Natural killer cells antigen CD94-like	Down
*LOC106032503*	−3.37	6.31E−02	Pantetheinase-like	Down
*RNF32*	−3.35	2.72E−02	Ring finger protein 32	Down
*LOC106044953*	−3.34	1.28E−01	Endonuclease 8-like 2	Down
*EFCAB3*	−3.19	8.11E−02	EF-hand calcium binding domain 3	Down
*LOC106040279*	−3.12	4.47E−02	Lysophosphatidic acid receptor 6-like	Down
*LOC106048719*	−2.97	1.91E−01	Antigen WC1.1-like	Down
*LOC106040105*	−2.95	4.01E−02	Chloride channel protein ClC-Kb-like	Down
*LOC106048620*	−2.85	1.24E−01	Proproteinase E-like	Down

1 *Log2FC: log_2_ (fold change).*

The full list of DEGs is presented in Supplementary Table S3. Moreover, the DEGs were analyzed by hierarchical cluster analysis. The samples from the same group were clustered together (Supplementary Figure S1), and the heat map visually reflected the differences in gene expression patterns between the HEP and LEP groups (Supplementary Figure S2).

### 3.3 Functional enrichment of DEGs

To determine the functionality of the DEGs, we mapped them according to the GO database. All DEGs were categorized into the three main categories of GO classification, namely, biological processes, molecular functions, and cellular components. In HEP and LEP comparison groups, the 404 DEGs were enriched to 703 GO terms, which included 586 biological processes, 90 molecular functions, and 28 cellular components (all enriched GO terms were presented in Supplementary Table S4). After the hierarchical clustering of biological processes GO terms, the top 10 GO clusters of all DEGs are listed in Table 3. The top three GO clusters were “cell fate commitment (GO:0045165),” “sensory organ development (GO:0007423),” and “positive regulation of protein phosphorylation (GO:0001934).”

**Table 3:** The top 10 clusters with their representative enriched GO (biological processes) terms across differentially expressed genes.

Cluster^1^	GO ID	Description	LogP^2^	Count^3^
1_Represent	GO:0045165	Cell fate commitment	−11.55	17
2_Represent	GO:0007423	Sensory organ development	−9.14	21
3_Represent	GO:0001934	Positive regulation of protein phosphorylation	−5.71	19
4_Represent	GO:0015850	Organic hydroxy compound transport	−5.68	9
5_Represent	GO:0048863	Stem cell differentiation	−5.64	9
6_Represent	GO:0050863	Regulation of T cell activation	−5.58	13
7_Represent	GO:0043583	Ear development	−5.37	10
8_Represent	GO:0009074	Aromatic amino acid family catabolic process	−5.36	4
9_Represent	GO:0006820	Anion transport	−5.28	14
10_Represent	GO:0007548	Sex differentiation	−5.25	11

1 GO terms were collected and grouped into clusters based on the similarity of its members, and sub-trees with a similarity of > 0.3 are considered cluster. The most significant term in a cluster is selected to represent the cluster.

2 LogP: log_10_ (*P*-value).

3 Count: the number of genes in the ontology term.

**Figure 2:**
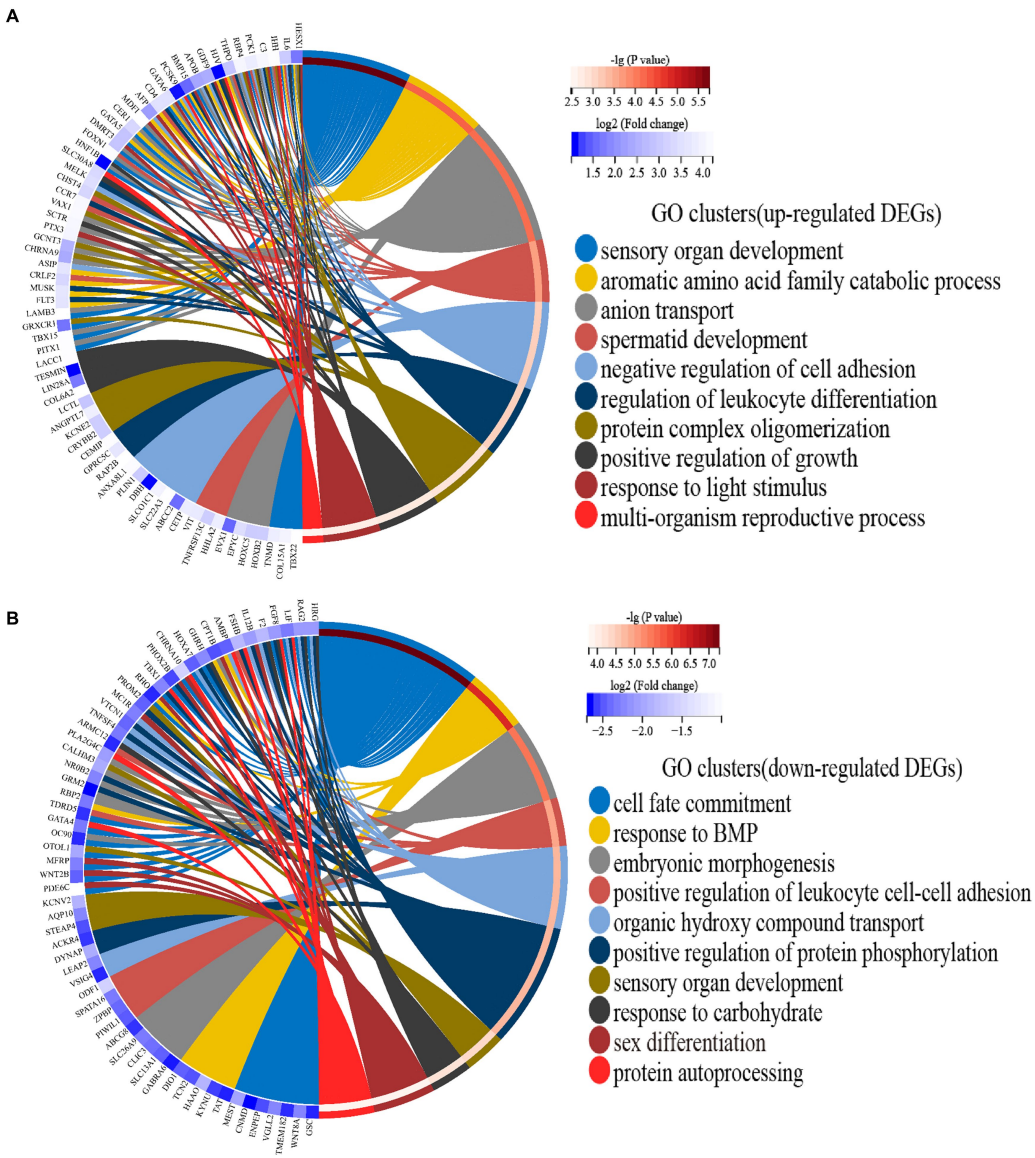
**(A)** Circos plots showing overlapping and specific responses of upregulated DEGs enrich the top 10 significant GO clusters. **(B)** Circos plots showing overlapping and specific responses of downregulated DEGs enrich the top 10 significant GO clusters. Note: The color depth of the outer circle of DEGs represents the log_2_ (fold change) indicating the gene expression level. The internal colorful ribbon represents the different GO clusters the DEGs enriched. DEG involvement in the GO clusters was identified by colored connecting lines.

The 191 upregulated genes were enriched to 244 GO terms, which included 191 biological processes, 37 molecular functions, and 16 cellular components. Moreover, the 213 downregulated genes were enriched to 363 GO terms, which included 298 biological processes, 47 molecular functions, and 18 cellular components. All enriched GO terms of upregulated and downregulated DEGs are presented in Supplementary Table S5. After hierarchical clustering of biological processes GO terms, the top 10 GO clusters of upregulated and downregulated DEGs are shown in Figure 2. The top three GO clusters of upregulated DEGs were “sensory organ development (GO:0007423),” “aromatic amino acid family catabolic process (GO:0009074),” and “anion transport (GO:0006820).” The top three GO clusters of downregulated DEGs were “cell fate commitment (GO:0045165),” “response to BMP (GO:0071772),” and “embryonic morphogenesis (GO:0048598).” Among these, “response to light stimulus (GO:0009416)” associated with the reproductive performance of geese attracted our attention. The genes and GO term members of the GO cluster, such as “response to light stimulus,” “sensory system development,” and “visual perception,” are listed in Table 4.

**Table 4:** GO term members of the two GO clusters associated with reproductive performance.

Cluster^1^	Description	Gene symbol
Represent	Response to light stimulus	*CPT1B, IL12B, MC1R, PDE6C, RHO, WNT2B, PHOX2B, MFRP, CHRNA10*
Member	Response to light stimulus	*CPT1B, IL12B, MC1R, PDE6C, RHO*
Member	Sensory system development	*PDE6C, RHO, WNT2B, PHOX2B, MFRP*
Member	Detection of external stimulus	*PDE6C, RHO, CHRNA10*
Member	Detection of abiotic stimulus	*PDE6C, RHO, CHRNA10*
Member	Retina development *in camera*-type eye	*PDE6C, RHO, MFRP*
Member	Eye morphogenesis	*PDE6C, WNT2B, MFRP*
Member	Response to radiation	*CPT1B, IL12B, MC1R, PDE6C, RHO*
Member	Camera-type eye development	*PDE6C, RHO, WNT2B, MFRP*
Member	Eye development	*PDE6C, RHO, WNT2B, MFRP*
Member	Visual system development	*PDE6C, RHO, WNT2B, MFRP*
Member	Visual perception	*PDE6C, RHO, MFRP*
Member	Sensory perception of light stimulus	*PDE6C, RHO, MFRP*

1 GO terms were collected and grouped into clusters based on the similarity of its members, and sub-trees with a similarity of > 0.3 are considered cluster. The most significant term in a cluster is selected to represent the cluster.

**Figure 3:**
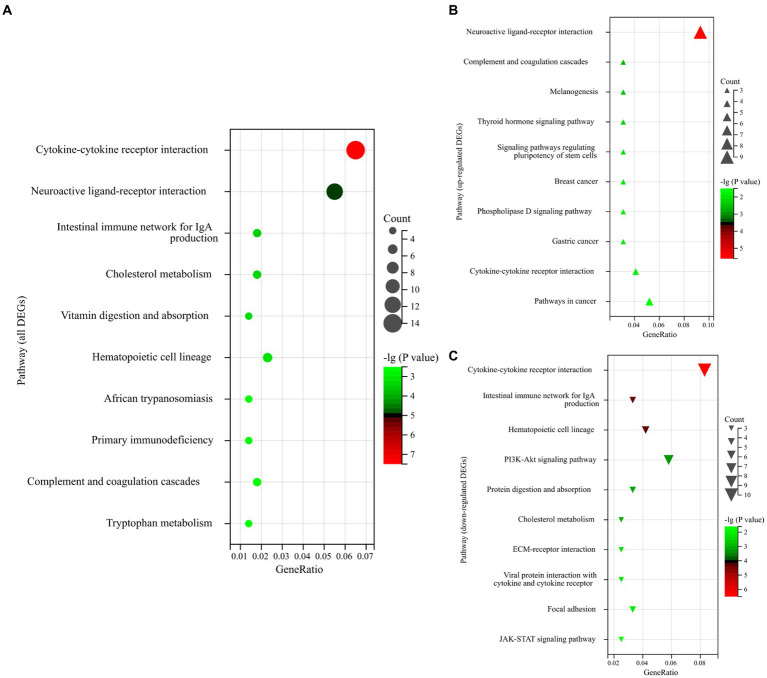
**(A)** Enrichment bubble plot of the top 10 ranked KEGG pathways of all DEGs. **(B)** Enrichment bubble plot of the top 10 ranked KEGG pathways of upregulated DEGs. **(C)** Enrichment bubble plot of the top 10 ranked KEGG pathways of downregulated DEGs. The size of the bubbles represents the number of assigned genes, and the color of the bubbles represents the -log (*p*-value). The greater the number of DEGs associated with the pathway, the larger the bubble.

To further understand the biological function of genes, a KEGG pathway analysis was performed for DEGs. In the first three pathways enriched by all DEGs, the KEGG pathways enriched significantly (*p*-value <0.05) and were mainly focused on “Cytokine-cytokine receptor interaction,” “Neuroactive ligand-receptor interaction,” and “Intestinal immune network for IgA production” (Figure 3A). More relevantly, most of the upregulated genes belong to the “Neuroactive ligand-receptor interaction” (Figure 3B). Regarding downregulated genes, most of the downregulated genes belong to the “Cytokine-cytokine receptor interaction” (Figure 3C).

### 3.4 Protein–protein interaction (PPI) network analysis

**Figure 4:**
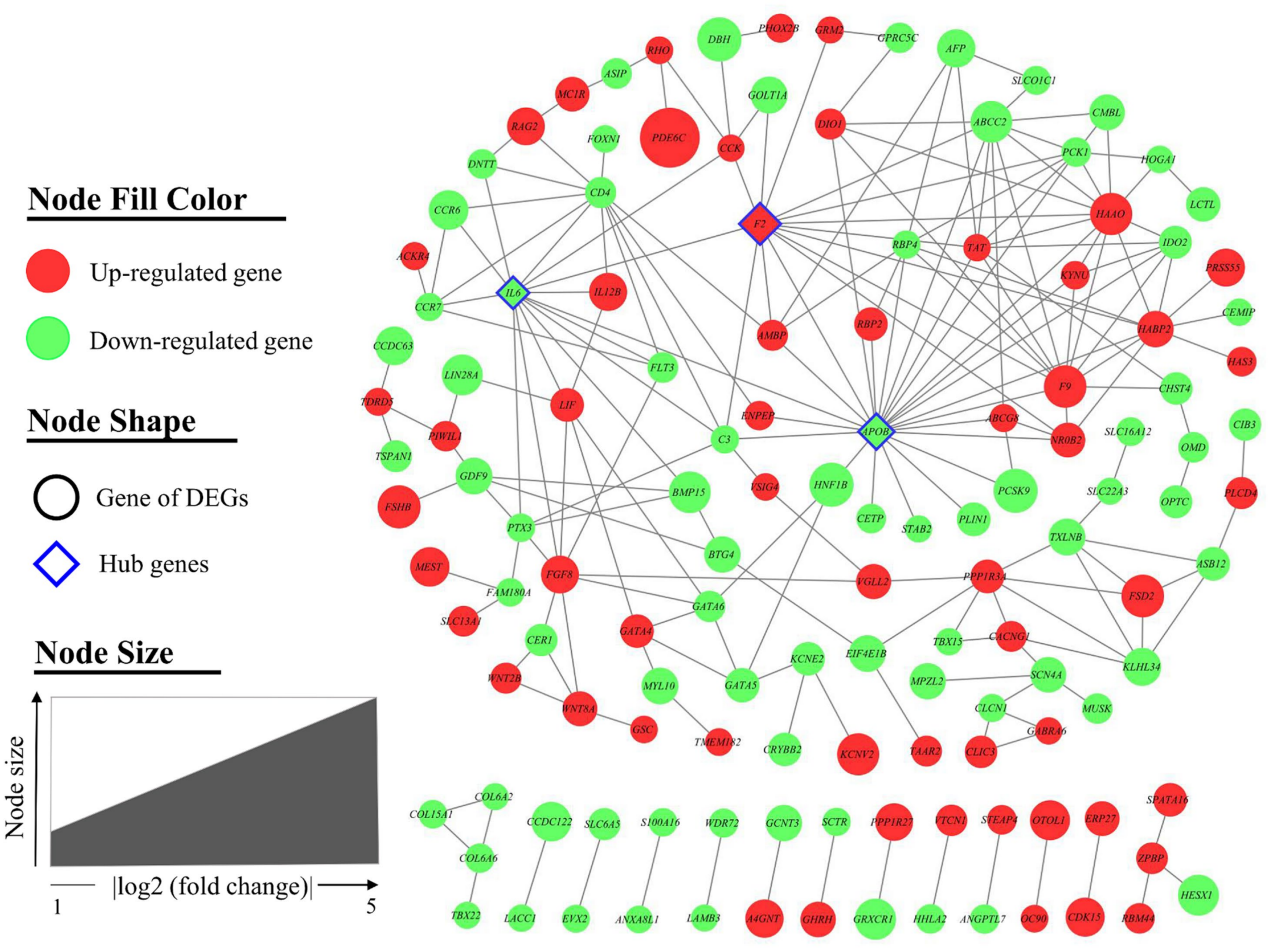
Protein–protein interaction (PPI) network of differentially expressed genes (DEGs). A total of 132 nodes and 210 interaction associations were identified. Nodes are filled with red for upregulated genes and green for downregulated genes. The node of the hub genes is diamond-shaped with a blue border. Node size indicated the fold change of each gene.

**Figure 5:**
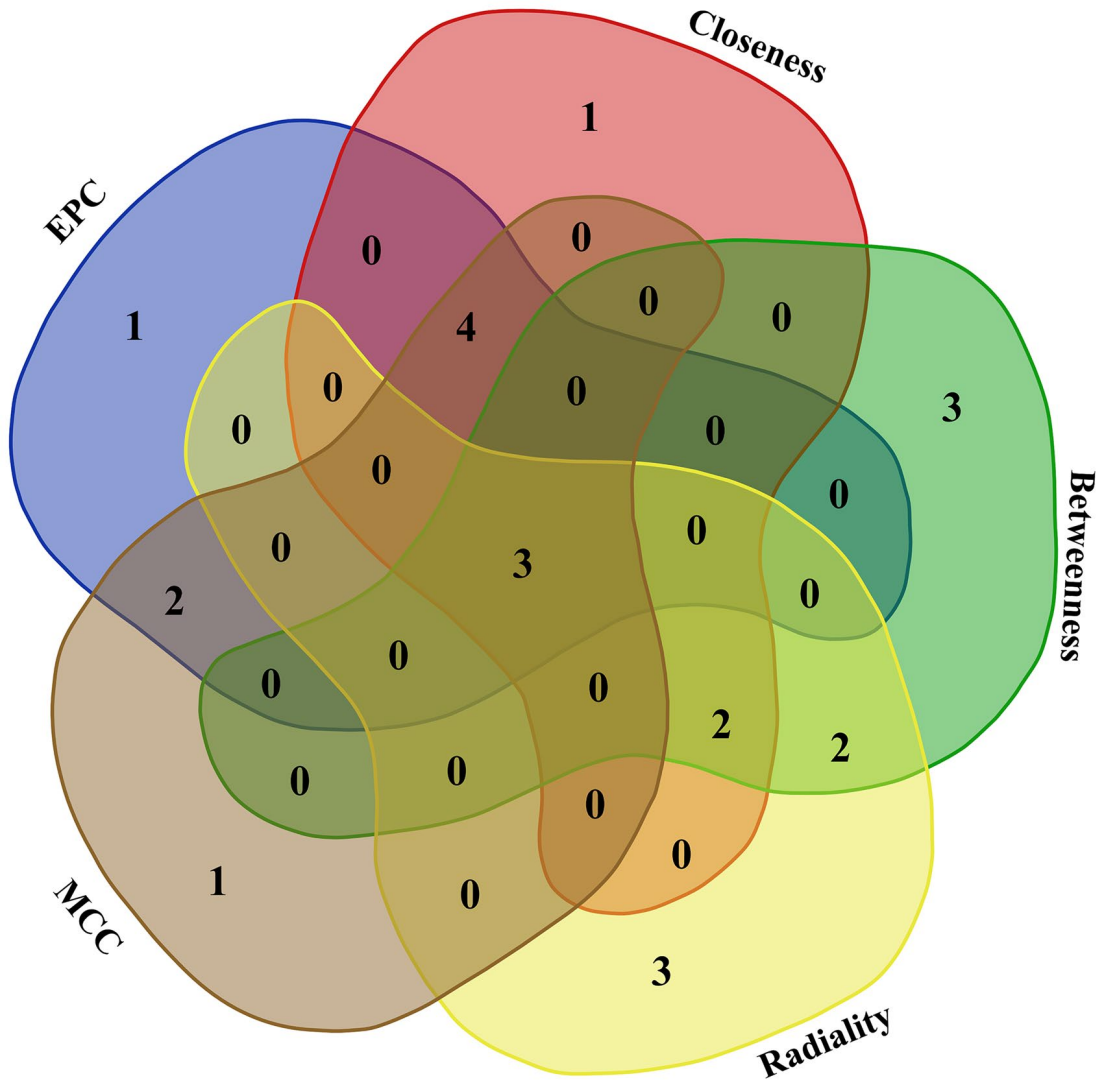
Venn plot to identify hub genes generated by five centrality methods (EPC, closeness, betweenness, radiality, and MCC). Different colored areas correspond to different centrality algorithms. The cross areas indicate the commonly accumulated DEGs, which include three hub genes (*F2*, *APOB*, and *IL6*).

The interaction between the proteins encoded by DEGs was further analyzed. A schematic diagram of the PPI network, including 132 nodes and 209 edges, is shown in Figure 4. Moreover, the top 10 genes evaluated by 5 centrality methods are listed in Table 5. We observed the intersections of five centrality methods and generated a Venn plot (Figure 5) to identify the hub genes of the PPI network. A total of three hub genes, namely, *F2* (coagulation factor II, thrombin), apolipoprotein B (*APOB*), and interleukin 6 (*IL6*), were identified.

**Table 5:** The top 10 genes evaluated by five centrality methods in the protein–protein interaction (PPI) network.

Rank	Gene	EPC score	Gene	Closeness score	Gene	Betweenness score	Gene	Radiality score	Gene	MCC score
1	*APOB*	46.74	*APOB*	48.36	*IL6*	3848.39	*IL6*	7.79	*APOB*	415
2	*F2*	46.58	*IL6*	45.78	*APOB*	3,243	*APOB*	7.63	*HAAO*	368
3	*F9*	46.58	*F2*	43.92	*PPP1R3A*	2,827	*C3*	7.60	*F2*	301
4	*HAAO*	46.56	*C3*	39.60	*FGF8*	2,765	*FGF8*	7.60	*ABCC2*	273
5	*TAT*	46.43	*F9*	38.82	*VGLL2*	2,598	*F2*	7.56	*F9*	272
6	*ABCC2*	46.30	*HAAO*	38.74	*F2*	1718	*PTX3*	7.43	*TAT*	267
7	*IL6*	46.00	*FGF8*	38.72	*CACNG1*	1,143	*LIF*	7.42	*PCK1*	130
8	*HABP2*	45.85	*HABP2*	37.24	*C3*	1,134	*BMP15*	7.33	*HABP2*	81
9	*PCK1*	45.84	*ABCC2*	37.07	*PTX3*	1,036	*FLT3*	7.32	*IDO2*	72
10	*RBP4*	45.25	*CD4*	37.04	*CCK*	993	*CCK*	7.30	*IL6*	38

**Figure 6:**
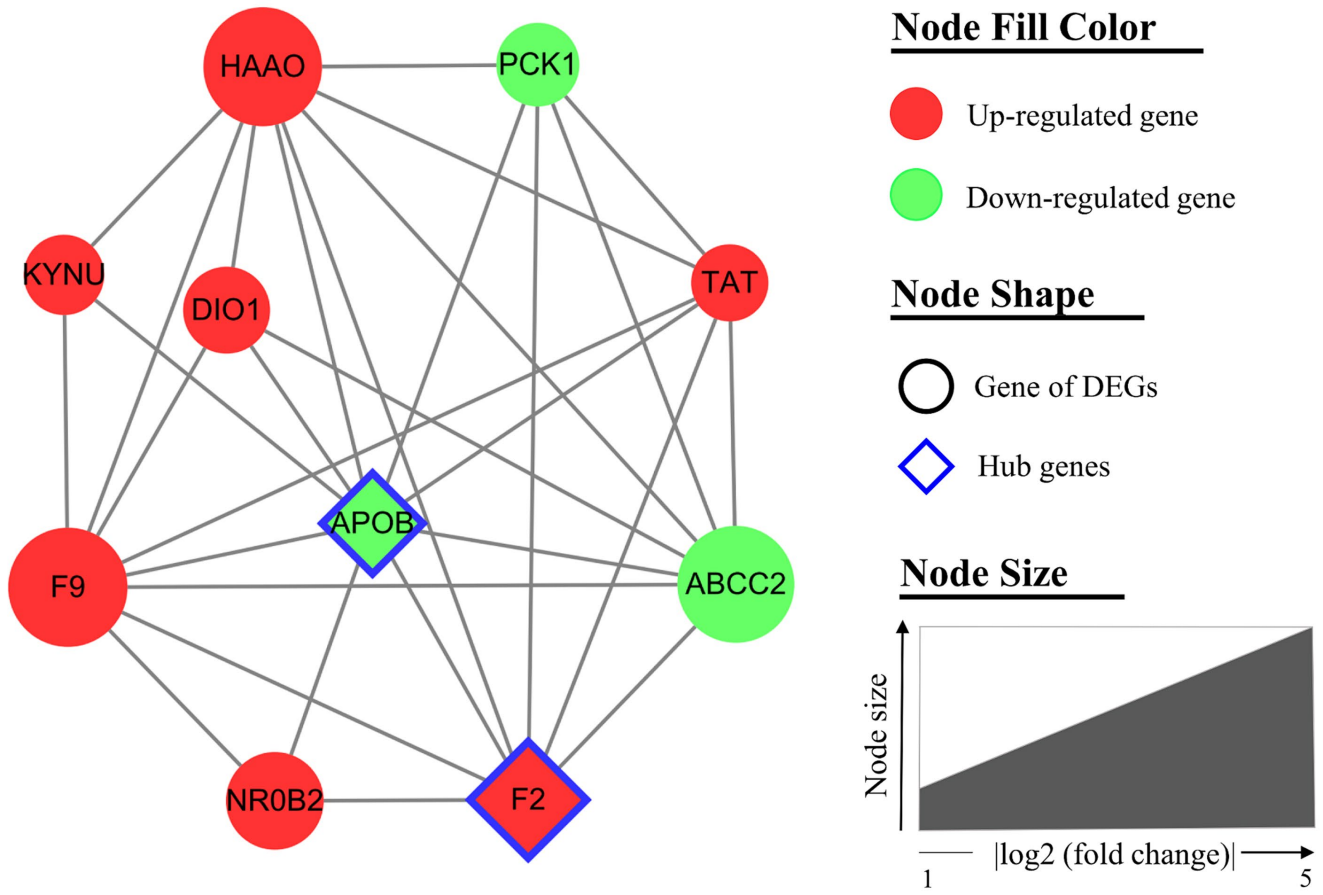
The hub module of the protein–protein interaction (PPI) network. The hub module (MCODE score = 6.67) includes 10 nodes and 30 interactions. Nodes are filled with red for upregulated genes and green for downregulated genes. The node of the hub genes is diamond-shaped with a blue border. Node size indicated the fold change of each gene.

Furthermore, the hub module (including 10 nodes and 30 interactions) of the PPI network was generated by MCODE (Figure 6). All genes of the hub module were analyzed by biological functional enrichment analysis (Table 6). The hub module is significantly enriched in the ‘aromatic amino acid family catabolic process’, ‘organic hydroxy compound metabolic process’, and ‘endoplasmic reticulum lumen’.

**Table 6:** Biological function enrichment analysis of the hub module network.

Cluster^1^	Category^2^	Description	LogP^3^	Symbols
1_Represent	GO (BP)	Aromatic amino acid family catabolic process	−7.84	*TAT, KYNU, HAAO, PCK1, DIO1, ABCC2*
2_Represent	GO (BP)	Organic hydroxy compound metabolic process	−4.95	*APOB, DIO1, PCK1, NR0B2, ABCC2*
3_Represent	GO (CC)	Endoplasmic reticulum lumen	−3.91	*APOB, F2, F9, PCK1, HAAO*
4_Represent	GO (BP)	Response to inorganic substance	−3.25	*APOB, TAT, HAAO, PCK1*

1 GO terms were collected and grouped into clusters based on the similarity of their members, and sub-trees with a similarity of > 0.3 are considered cluster. The most significant term in a cluster is selected to represent the cluster.

2 BP: biological process; CCs: cellular components.

3 LogP: log_10_ (*P*-value).

Gene set enrichment analysis (GSEA).

**Figure 7:**
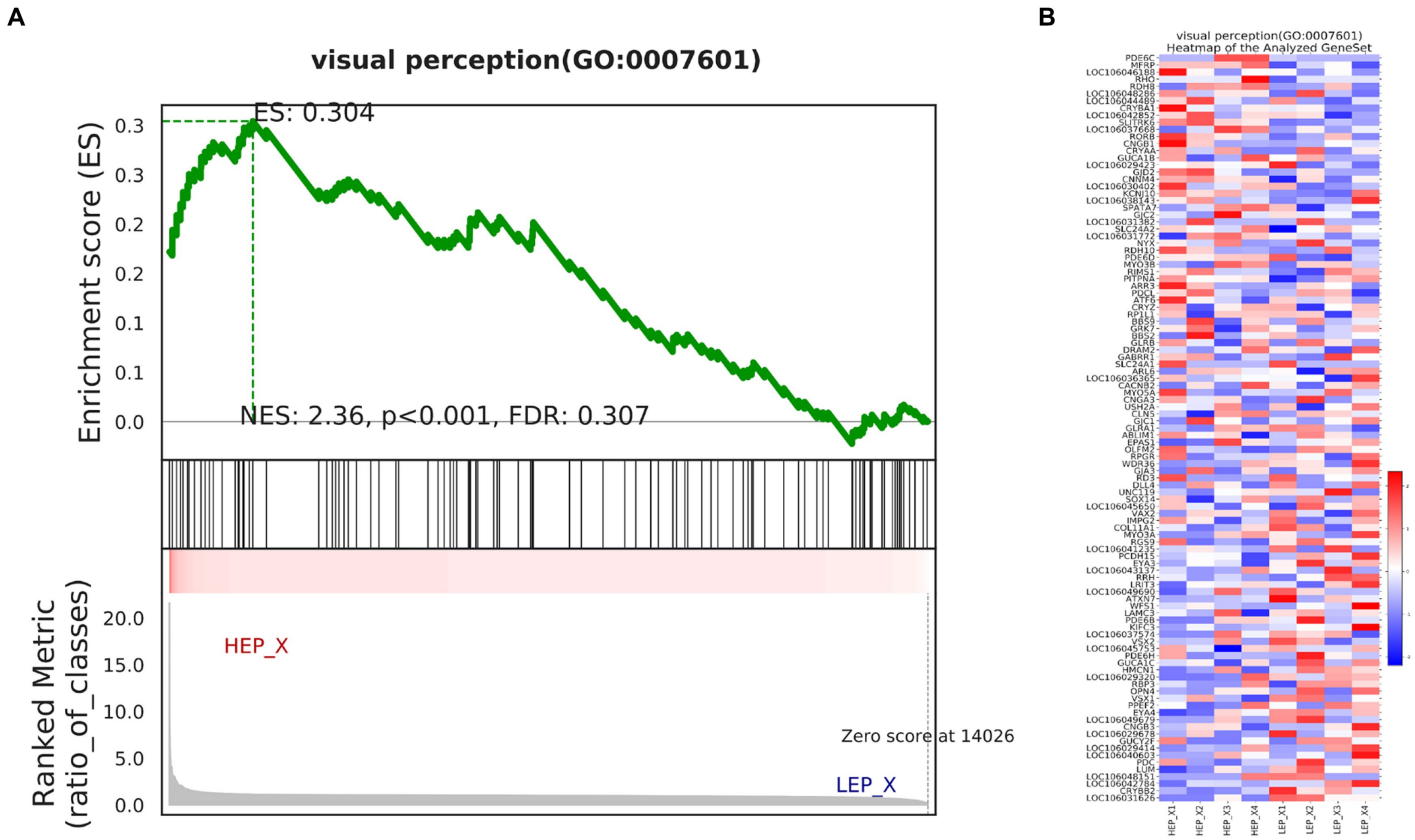
**(A)** Enrichment of genes in the VISUAL PERCEPTION (GO:0007601) by gene set enrichment analysis (GSEA). **(B)** The heat map of core enrichment genes in the gene set visual perception (GO:0007601). The enrichment plots contain profiles of the running enrichment scores (ES) and positions of gene set members on the rank-ordered list in GSEA.

We further assessed the function of all genes using a GSEA. GSEA was performed using a GO-based list, including 9,996 gene sets. The results of GSEA are presented in Supplementary Table S6. The top 10 GO-based gene sets with positive and negative NES values were selected and are shown in Table 7. Positive and negative NES indicate higher and lower expression in HEP geese, respectively. The higher expression gene sets in HEP geese were involved in ‘myelin sheath’, ‘serine-type endopeptidase activity’, and ‘cytokine activity’. The lower expression gene sets in HEP geese were involved in ‘collagen-containing extracellular matrix’, ‘extracellular matrix structural constituent conferring tensile strength’, and ‘extracellular matrix structural constituent’. Among these, the gene set of ‘visual perception’ (NES = 2.36, *p* < 0.001) caught our attention (Figure 7).

**Table 7:** Gene set enrichment analysis (GSEA) of HEP and LEP geese.

Gene set	NES^1^	*P*-value	Higher expression in HEP or LEP^2^
Myelin sheath (GO:0043209)	2.70	<0.001	HEP
Serine-type endopeptidase activity (GO:0004252)	2.58	<0.001	HEP
Cytokine activity (GO:0005125)	2.54	0.001	HEP
Heme binding (GO:0020037)	2.37	0.001	HEP
Tricarboxylic acid cycle (GO:0006099)	2.36	<0.001	HEP
Visual perception (GO:0007601)	2.36	<0.001	HEP
Cell fate commitment (GO:0045165)	2.31	0.003	HEP
Axonogenesis (GO:0007409)	2.29	0.001	HEP
Schaffer collateral - CA1 synapse (GO:0098685)	2.24	0.003	HEP
Potassium ion transmembrane transport (GO:0071805)	2.24	0.001	HEP
Ribonucleoprotein complex (GO:1990904)	−2.24	<0.001	LEP
Collagen binding (GO:0005518)	−2.26	<0.001	LEP
Muscle alpha-actinin binding (GO:0051371)	−2.28	<0.001	LEP
Cornification (GO:0070268)	−2.30	<0.001	LEP
Basement membrane (GO:0005604)	−2.41	<0.001	LEP
Collagen trimer (GO:0005581)	−2.47	<0.001	LEP
Spliceosomal complex (GO:0005681)	−2.66	<0.001	LEP
Extracellular matrix structural constituent (GO:0005201)	−2.82	<0.001	LEP
Extracellular matrix structural constituent conferring tensile strength (GO:0030020)	−2.96	<0.001	LEP
Collagen-containing extracellular matrix (GO:0062023)	−3.63	<0.001	LEP

1 NES: normalized enriched score.

2 Positive and negative NES indicate higher and lower expression in HEP, respectively.

### 3.5 Validation of RNA-seq results

**Figure 8:**
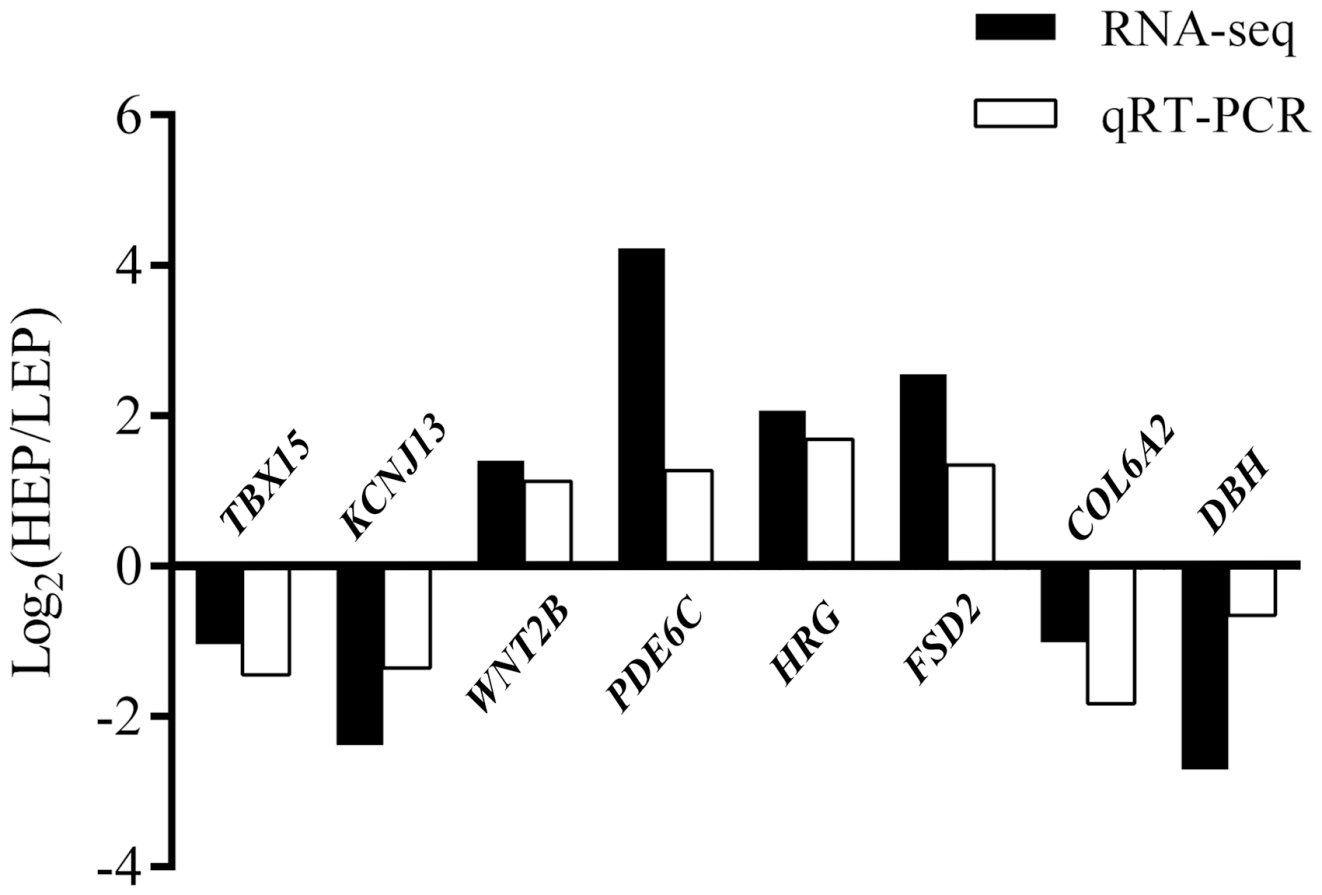
Evaluation and comparison of mRNA expression levels (Log2FC) of eight DEGs when analyzed by RNA-seq and quantitative qRT-PCR. The relative expression levels of genes were calculated according to the 2^−ΔΔ Ct^ method using *β*-actin as an internal reference RNA.

To verify RNA-seq results, a total of eight DEGs were selected for qRT-PCR analysis: *TBX15*, *KCNJ13*, *WNT2B*, *PDE6C*, *HRG*, *FSD2*, *COL6A2*, and *DBH* (Figure 8). Our validation results showed that the eight DEGs had the same expression trends in RNA-seq and qRT-PCR, which validated their accuracy.

## 4 Discussion

Although the research on the egg production performance of poultry has not been interrupted, its molecular mechanism is still unclear, especially in geese. In this study, we used RNA-seq to detect the hypothalamic transcriptome profiles of four geese with HEP and four geese with LEP. Egg production performance is a low heritability trait that is regulated by multiple genes (13). In the function analysis of DEGs, we found that HEP geese upregulated genes related to “response to light stimulus,” “sensory system development,” and “visual perception” (Table 4 and Figure 2). Coincidentally, GSEA results also showed that HEP geese upregulated many genes involved in “visual perception” (Figure 7).

For animals, vision is not just for subjective observation of their surroundings. More importantly, it sends unconscious visual information to the brain. The transmission of visual information to the hypothalamus is called the retinal hypothalamic tract (RHT), which plays an important role in neuro-endocrine control of diurnal and longer rhythms (20). For example, compared with the new moon, many birds sleep a few hours less at the full moon, and artificial light at night has a similar sleep-suppressing effect (21). More relevantly, many activities of vertebrates, including reproductive behavior, depend on daily and seasonal light cues (22). Over the past decade, it has been well established that light has an important effect on the egg production performance of geese (12). A previous study in Yangzhou geese indicated that the use of white or red monochrome lamps (long photoperiod applied for 11 h per day) can maintain reproductive system function for a longer period, thus improving egg production performance (23). Moreover, it was well documented in Hungarian white geese that increasing the photoperiod allowed the geese to continue reproductive activity for a longer time, resulting in higher egg production performance (24). In addition to reproductive performance, light also has an important effect on the growth performance of Magang geese (25). According to the aforementioned results, it could be hypothesized that visual perception plays an important role in the regulation of egg production in geese.

In this study, we found three DEGs, namely, *PDE6C, RHO*, and *MFRP*, were involved in “visual perception” (Table 4). Moreover, *PDE6C* was the most altered (upregulated) gene in HEP geese. *PDE6* is an effector in the phototransduction cascade in rods and cones (26), and the rod *PDE6* catalytic core is a heterodimer of *PDE6A* and *PDE6B* subunits, while the conical *PDE6C* subunit forms a catalytic homodimer (27). Rods and cones of photoreceptors convert visible light into nerve signals that are eventually transmitted to the brain (28). A number of studies have shown that the mutation in *PDE6C* will affect both cone and rod photoreceptors and eventually cause retinal degenerative diseases, such as achromatopsia in humans (29) and retinal degeneration in zebrafish (30). In essence, *PDE6C* encodes the catalytic alpha subunit of cone photoreceptor phosphodiesterase, a key regulatory element of cone phototransduction (31). Rhodopsin (*RHO*) is a photosensitive pigment in the retina (32). RHO is the photoreceptor in rod-shaped photoreceptor cells, which is the origin of dark vision (33). Interestingly, a previous study indicated genes related to phototransduction and photoreceptor development in the retina of domestic chickens, including *RHO* and *PDE6B*, have experienced positive selection and downregulated expression in domestic chickens during evolution, resulting in weaker night vision in domestic chickens than Red Junglefowl (34). *MFRP* gene encodes type II transmembrane protein, which is mainly expressed in retinal pigment epithelial and ciliary bodies (35). It was well documented that mutations in the *MFRP* gene cause a variety of vision disorders, including hyperopia, acute angle-closure glaucoma, retinitis pigmentosa, retinal folds, macular edema, retinal cysts, and retinal degeneration (36).

In the current study, *PDE6C, RHO*, and *MFRP* were upregulated in the hypothalamus of HEP geese. Birds’ skulls and brains are highly transparent, and because of that the sunlight is scattered and absorbed by the covering tissue, a large number of photons penetrate deep into the brain and stimulate these deep brain photoreceptors (DBPs) (37). A previous study indicated that extraretinal photoreceptors are located in the medial-basal hypothalamus (MBH) and regulate the photoperiod control of seasonal reproduction in birds (38). It was well documented that LH release can be achieved by electrical stimulation of MBH (39), while GnRH can be secreted by the stimulation of MBH preserved *in vitro* by prolonged illumination (40). Collectively, it is logical to assume that upregulated genes related to visual perception in the hypothalamus may regulate poultry neuroendocrine hormones by affecting peripheral photoreceptors in MBH, thus leading to differences in egg production. In this scenario, it can be inferred that *PDE6C*, *RHO*, and *MFRP* were the key candidate genes for the geese egg production trait. Moreover, our results and further conjecture provide the anatomical substrate for the artificial light regulation of egg production in geese.

It is worth signaling that the previous studies on the molecular regulation mechanism of light on geese’s egg production performance basically used different light conditions to stimulate the difference in egg production and then analyzed the molecular regulation (transcriptome) difference (12, 23–25). In such a situation, the differences in genes and pathways obtained in these articles are essentially caused by different light stimuli rather than different egg production, that is, these gene changes may not have a causal relationship with the change in geese’s egg production, but rather due to differences in the regulatory mechanisms by which geese themselves respond to different light stimuli. Consequently, previous results are useful for optimizing lighting management during geese laying periods but might not be suitable for screening candidate genes for marker-assisted selection. To the best of our knowledge, our study is the first to observe that geese with different egg productions show differences in response to light stimulation and visual perception under the same environmental conditions.

In this study, the analysis of the PPI network showed that *F2*, *APOB*, and *IL6* were hub genes among the DEGs. *F2* (coagulation factor II, thrombin) encodes the prothrombin protein, which plays a significant role in response to vascular injury by being converted into thrombin (41). Thrombin is crucial for activating platelets and increasing endothelial permeability, which prevents blood loss and promotes vascular remodeling (42, 43). Interestingly, higher levels of *F2* mRNA have been detected in neurons and glial cells in the central nervous system, suggesting a potential but unexplored role in neurological functions (44).

*APOB*, a major protein component of chylomicrons and VLDL, is involved in lipid transport and the formation of egg yolks in poultry (45, 46). Previous studies have shown that APOB expression is higher in the liver of high egg-laying chickens compared to the low egg-laying chickens, indicating its role in reproductive performance (47). However, high *APOB* expression in the ovaries may lead to excessive fat deposition, potentially damaging ovulation (48). The role of *APOB* in the hypothalamus remains unclear, although it has been noted to be upregulated during cold stress in chickens, suggesting a possible link to environmental stress responses (49). IL6 is a multifunctional cytokine involved in immune response regulation and has been associated with neuroinflammation (50–52). In this study, lower levels of *APOB* and IL6 expression were observed in HEP geese compared to LEP geese, which may reflect a higher susceptibility to cold stress and neuroinflammation in LEP geese during the laying period. In summary, these findings highlight the potential roles of *F2, APOB*, and *IL6* in the regulation of egg production in geese. Further research is necessary to elucidate the specific mechanisms by which these genes influence reproductive performance and how they interact with environmental factors.

Although this study provides valuable insights into the genetic and environmental factors influencing egg production in geese, several limitations should be acknowledged. The sample size was relatively small, which may limit the generalizability of the findings. Future studies with larger sample sizes are needed to confirm these results. Although we monitored and controlled for many potential confounding factors, there may still be unrecognized variables that could influence the outcomes. The study focused primarily on genetic and phenotypic data, and further research incorporating molecular and biochemical analyses would provide a more comprehensive understanding of the mechanisms underlying egg production in geese.

In summary, the transcriptome profile differences between the hypothalamus of HEP and LEP geese were analyzed in detail. Function enrichment, interaction analysis, and GSEA methods were performed to further analyze the changes in biological processes between different egg production geese. GO annotations indicated that DEGs related to egg production were mainly enriched in biological processes such as “response to light stimulus,” “sensory system development,” and “visual perception.” Our results demonstrated that visual perception plays a crucial role in the regulation of egg production in geese. Six potential candidate genes (*PDE6C, RHO*, *MFRP, F2, APOB*, and *IL6*) were identified based on their corresponding GO terms and interaction network. These candidate genes can be used as selection markers to improve the egg production of Wanxi white geese. This study confirmed the causal relationship between visual perception and egg production, providing a research basis and reference data for future studies on the regulatory mechanisms of environmental light on egg production in geese. These findings not only enhance our understanding of the genetic basis of egg production in geese but also offer valuable insights for breeding strategies aimed at increasing egg yield. By integrating genetic data with practical breeding approaches, we can better address the challenges in goose production and contribute to the sustainable development of the poultry industry.

## Data Availability

The data presented in the study are deposited in the National Center for Biotechnology Information repository, accession number: PRJNA846331.

## Footnotes

^1^ http://genome.ucsc.edu/

^2^ http://metascape.org

^3^ http://string-db.org/

^4^ http://www.cytoscape.org/

^5^ http://bioinformatics.psb.ugent.be/webtools/Venn/

^6^ http://www.broad.mit.edu/gsea/index.html

^7^ https://www.ncbi.nlm.nih.gov/tools/primer-blast/

## Supplementary material

Supplementary Table S1 Quality metrics of transcripts in the hypothalamus of HEP and LEP geese.

Supplementary Figure S1 The principal component analysis of transcripts in the hypothalamus of HEP and LEP geese.
