# Supplementary figures and images for: The hypothalamic transcriptome reveals the importance of visual perception on the egg production of Wanxi white geese
Source: Front Vet Sci. 2024 Sep 20;11:1449032. doi: 10.3389/fvets.2024.1449032 (PMC11450866; doi:10.3389/fvets.2024.1449032)

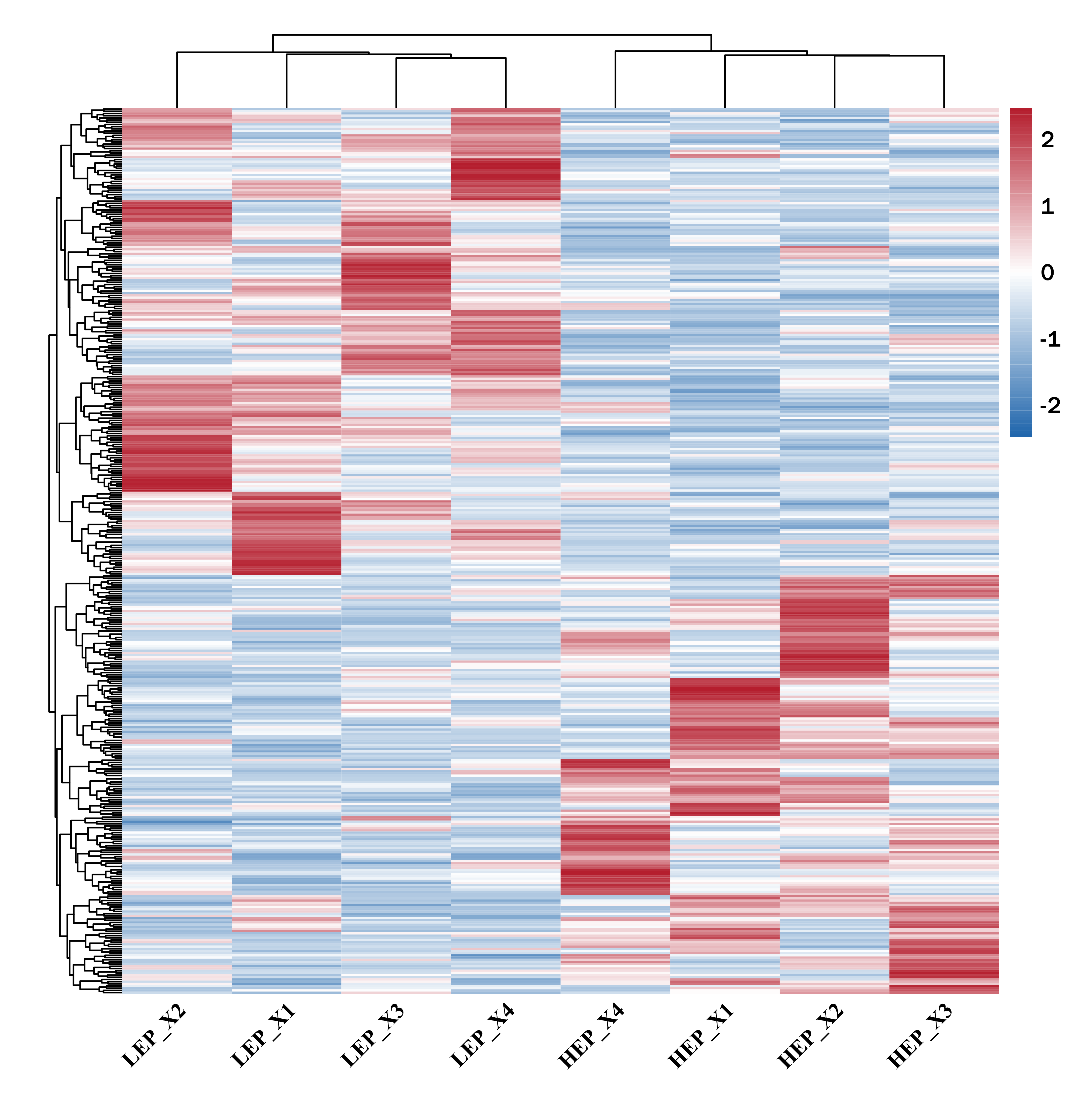

Supplement: Supplementary Figure S1 — The principal component analysis of transcripts in the hypothalamus of HEP and LEP geese. [file Image_1.TIF]

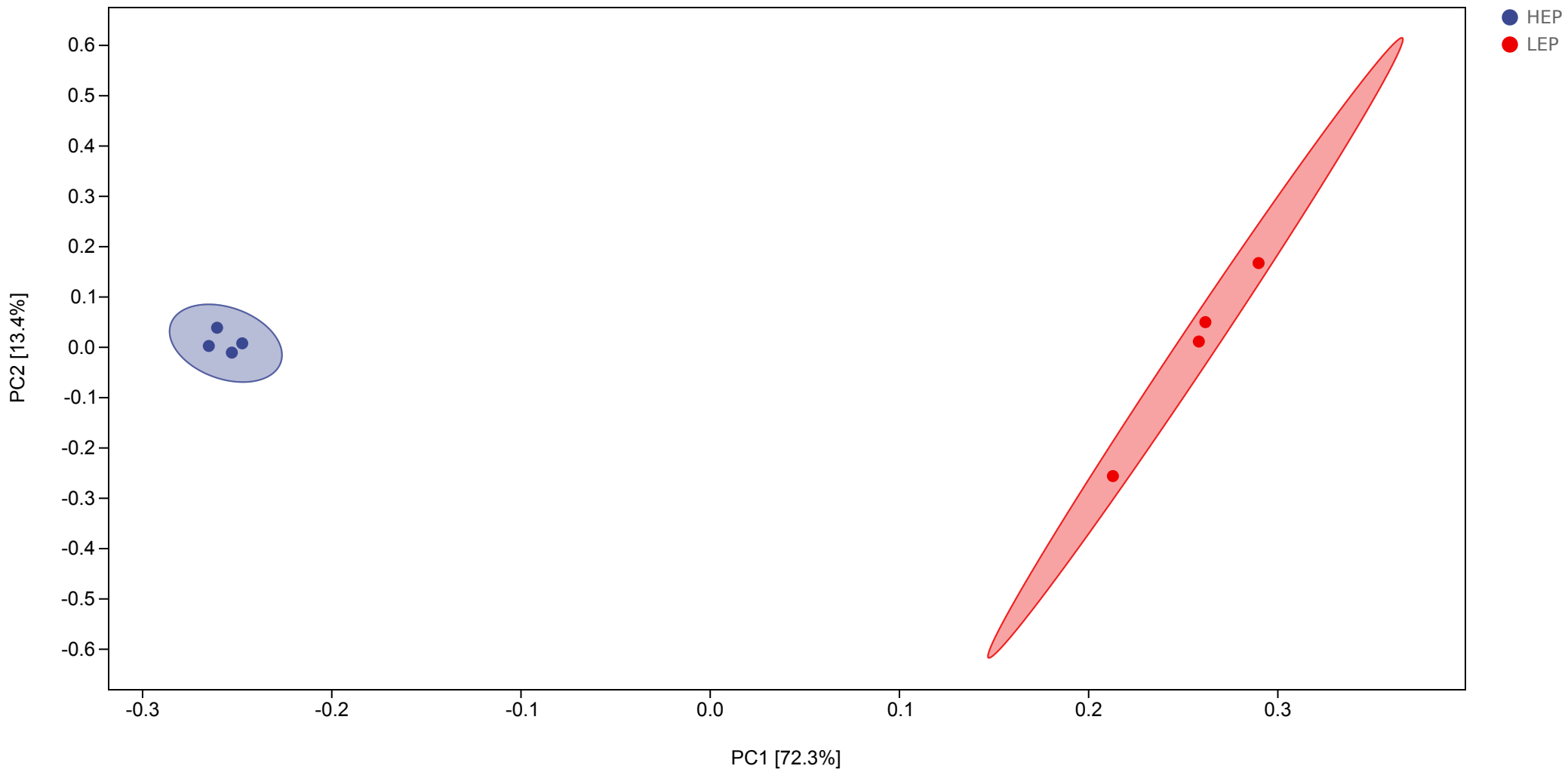

Supplement: Supplementary file 8 [file Data_Sheet_1.PDF]
